# Di-(2-ethylhexyl) Phthalate Enhances Atopic Dermatitis-Like Skin Lesions in Mice

**DOI:** 10.1289/ehp.8985

**Published:** 2006-05-15

**Authors:** Hirohisa Takano, Rie Yanagisawa, Ken-ichiro Inoue, Takamichi Ichinose, Kaori Sadakane, Toshikazu Yoshikawa

**Affiliations:** 1 Environmental Health Sciences Division, National Institute for Environmental Studies, Tsukuba, Japan; 2 Inflammation and Immunology, Kyoto Prefectural University of Medicine, Kyoto, Japan; 3 Department of Health Sciences, Oita University of Nursing and Health Sciences, Oita, Japan

**Keywords:** atopic dermatitis, chemokines, di-(2-ethylhexyl) phthalate, eosinophils, mast cells

## Abstract

Di-(2-ethylhexyl) phthalate (DEHP) has been widely used in polyvinyl chloride products and has become ubiquitous in the developed countries. DEHP reportedly displays an adjuvant effect on immunoglobulin production. However, it has not been elucidated whether DEHP is associated with the aggravation of atopic dermatitis. We investigated the effects of DEHP on atopic dermatitis-like skin lesions induced by mite allergen in NC/Nga mice. NC/Nga male mice were injected intradermally with mite allergen on their right ears. In the presence of allergen, DEHP (0, 0.8, 4, 20, or 100 μg) was administered by intraperitoneal injection. We evaluated clinical scores, ear thickening, histologic findings, and the protein expression of chemokines. Exposure to DEHP at a dose of 0.8–20 μg caused deterioration of atopic dermatitis-like skin lesions related to mite allergen; this was evident from macroscopic and microscopic examinations. Furthermore, these changes were consistent with the protein expression of proinflammatory molecules such as macrophage inflammatory protein-1α (MIP-1α) and eotaxin in the ear tissue in overall trend. In contrast, 100 μg DEHP did not show the enhancing effects. These results indicate that DEHP enhances atopic dermatitis-like skin lesions at hundred-fold lower levels than the no observed adverse effect level determined on histologic changes in the liver of rodents. DEHP could be at least partly responsible for the recent increase in atopic dermatitis.

The prevalence of allergic diseases has rapidly increased in developed countries throughout the past several decades (Beasley et al. 2003). Changes in environmental factors such as allergen load, infectious disease profile, vaccination, and the presence of environmental adjuvants, rather than genetic factors, are likely to be considered as the cause of this increase (Etzel 2003; Strachan 2000). We have previously reported that diesel exhaust particles—environmental adjuvants that contain a vast number of organic chemicals—enhance murine models of allergic asthma (Ichinose et al. 2004; Miyabara et al. 1998; Sadakane et al. 2002; Takano et al. 1997). A recent epidemiologic study has revealed the positive association between allergic asthma in children and phthalate esters in house dust (Bornehag et al. 2004). Among phthalate esters, di-(2-ethylhexyl) phthalate (DEHP) has been widely used [1.8 million metric tons/year (Cadogan and Howick 1996)] in polyvinyl chloride products, including vinyl flooring, wall coverings, food containers, and infant toys, and has become ubiquitous in developed countries since the end of World War II. Larsen et al. (2001) suggested that DEHP displays an adjuvant effect on allergen-related immunoglobulin production. In contrast, house dust mite allergens—major allergens in humans—closely relate to (Schafer et al. 1999) or enhance (Sanda et al. 1992) atopic dermatitis. However, the association between DEHP and atopic dermatitis has never been elucidated. In the present study we investigated the effects of DEHP on atopic dermatitis-like skin lesions induced by mite allergen in NC/Nga mice, an animal model described previously by Sasakawa et al. (2001).

## Materials and Methods

### Animals

Seven-week-old SPF NC/Nga male mice (22–25 g body weight) were purchased from Charles River Japan (Osaka, Japan) and maintained in conventional conditions for 1 week. They were fed a commercial diet (CE-2; Japan Clea Co., Tokyo, Japan) and water *ad libitum*. Mice were housed in an animal facility that was maintained at 22–26°C with 40–69% humidity and a 12 hr/12 hr light/dark cycle. The study adhered to the U.S. National Institutes of Health guidelines for the use of experimental animals (Institute of Laboratory Animal Resources 1996). The mice were handled according to the National Institute for Environmental Studies animal guidelines. Animals were treated humanely and with regard for alleviation of suffering.

### Study protocol

Mice were divided into seven experimental groups. One group received no treatment (nontreated). In the other groups, mice were treated with 10 μL saline or 5 μg mite allergen extract [*Dermatophagoides pteronyssinus* (Dp); Cosmo Bio LSL, Tokyo, Japan] dissolved in saline; mice were injected intradermally on the ventral side of their right ears on days 0, 2, 4, 7, 9, 11, 14, and 16 under anesthesia with 4% halothane (Takeda Pharmaceutical Company, Ltd., Osaka, Japan). In the presence of allergen, DEHP at a dose of 0, 0.8, 4, 20, or 100 μg dissolved in 0.1 mL of olive oil (vehicle) was administered by intraperitoneal injection on days –4, 3, 10, and 17. Twenty-four hours after each intradermal injection, we measured ear thickness using a gauge (Ozaki Mfg, Osaka, Japan) and evaluated clinical scores by skin dryness, eruption, edema, and wound graded from 0 to 3 (no symptoms, 0; mild, 1; moderate, 2; and severe, 3). The clinical scores were estimated as the sum of these values.

### Histologic evaluation

Right ears of mice were removed 48 hr after the last injection of Dp (day 18) and were fixed in 10% neutral phosphate-buffered formalin (pH 7.2) and embedded in paraffin. Sections of 3 μm were routinely stained with hematoxylin and eosin (H&E) or with toluidine blue (pH 4.0). Histologic analyses were performed using an AX80 microscope (Olympus, Tokyo, Japan). We measured the length of the cartilages in each specimen and counted the numbers of eosinophils and mast cells in each sample using a video micrometer (VM-30; Olympus). The infiltration of eosinophils and mast cells were morphometrically evaluated as the number of cells per millimeter of the cartilages in a blind fashion. We also evaluated the degranulation of mast cells as not degranulated (0%), mildly degranulated (0–50%), and severely degranulated (> 50%).

### ELISA

Right ears of mice were removed 48 hr after the last injection of Dp (day 18) and were homogenized and centrifuged as previously described (Takano et al. 1997). We conducted enzyme-linked immunosorbent assays (ELISAs) for macrophage inflammatory protein-1α (MIP-1α) (R&D Systems, Minneapolis, MN, USA) and eotaxin (R&D Systems) in the ear tissue supernatants according to the manufacturer’s instructions. The detection limits of MIP-1α and eotaxin were 1.5 pg/mL and 3 pg/mL, respectively.

### Statistical analysis

Data are reported as mean ± SE. Differences among groups were determined using Dunnett’s multiple comparison test. A *p*-value < 0.05 was considered to be significant (StatView, version 5.0; Abacus Concepts Inc., Berkeley, CA, USA).

## Results

### DEHP enhances symptoms of atopic dermatitis-like skin lesions

**Figure 1:**
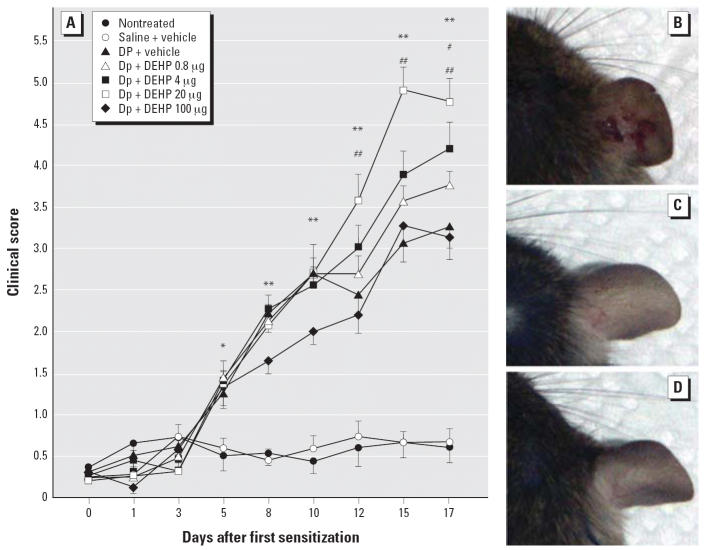
Exposure to DEHP exacerbates atopic dermatitis-like skin lesions induced by Dp on ears. (*A*) Clinical scores of the ears 24 hr after each injection (differences among groups were determined using Dunnett’s multiple comparison test; data are the mean ± SE of 16 animals per group). (*B–D*) Macroscopic features 48 hr after the last injection of Dp: (*B*) Dp + 20 μg DEHP, (*C*) Dp + vehicle, and (*D*) nontreated. *p < 0.05, Dp-treated groups vs. nontreated group and saline + vehicle group. **p < 0.01, Dp treated groups vs. nontreated group and saline + vehicle group. ^#^*p* < 0.05, Dp + 4 μg DEHP group vs. Dp + vehicle group. ^##^*p* < 0.01, Dp + 20 μg DEHP group vs. Dp + vehicle group.

To evaluate the effects of DEHP on atopic dermatitis-like skin lesions induced by Dp, we examined clinical scores and ear thickening. Treatment with Dp significantly enhanced clinical scores (*p* < 0.05: Figure 1A), including dryness, eruption, wound, edema, and ear thickening (*p* < 0.05; see Supplemental Material available online at http://www.ehponline.org/docs/2006/8985/suppl.pdf) compared with no treatment or saline from day 5. After day 12, exposure to DEHP at 0.8–20 μg dose-dependently increased clinical scores and ear thickening compared with vehicle exposure in the presence of allergen. The symptoms were most prominent on the treatment with DEHP at 20 μg (*p* < 0.01 vs. vehicle treatment). In particular, combined exposure to 20 μg DEHP and allergen caused marked wound (Figure 1B) compared with exposure to vehicle and allergen (Figure 1C). We observed no change in the nontreated group (Figure 1D) or the saline group (data not shown). On the other hand, 100 μg DEHP did not show the enhancing effects (Figure 1A).

### DEHP aggravates histologic changes in the skin related to Dp

**Figure 2:**
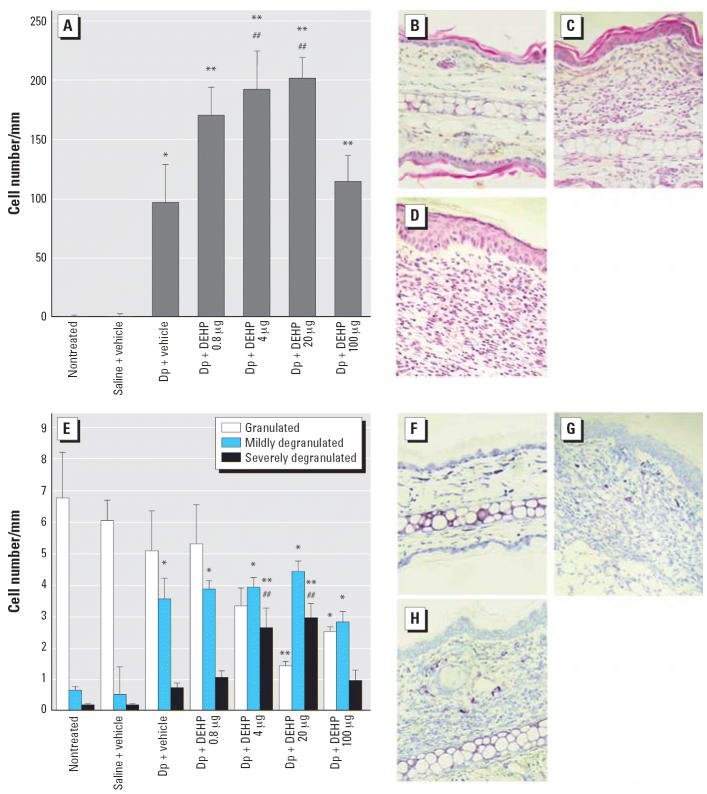
Histologic changes in the ear 48 hr after the last injection of Dp. The infiltration of eosinophils (*A*) and mast cells (*E*) was morphometrically evaluated as the number of cells per millimeter of cartilage, and the degranulation of mast cells was evaluated as nondegranulated (0%), mildly degranulated (0–50%), and severely degranulated (> 50%). Differences among groups were determined using Dunnett’s multiple comparison test; data shown are the mean ± SE of four animals per group. (*B*–*D, F–H*). Photomicrographs (400× magnification) of ear tissue from the nontreated group (*B*, *F*), Dp + vehicle group (*C*, *G*), and Dp + 20 μg DEHP group (*D*, *H*) are shown with H&E staining (*B–D*) or toluidine blue staining (*F–H*). *p < 0.05 vs. nontreated group and saline + vehicle group. **p < 0.01 vs. nontreated group and saline + vehicle group. ^##^*p* < 0.01 vs. Dp + vehicle group.

Histologic examination with H&E staining showed that exposure to 0.8–20 μg DEHP dose-dependently enhanced the infiltration of eosinophils into the skin lesion in the presence of allergen (Figure 2A; *p* < 0.01 for 4 or 20 μg DEHP vs. vehicle). In overall trend, these changes were paralleled by the severity of mast cell degranulation (Figure 2E). Treatment with 20 μg DEHP (Figure 2D,H) caused more prominent histologic changes than that with vehicle (Figure 2C,G) in the presence of allergen. No pathologic alterations were found in the nontreated group (Figure 2B,F) or saline treatment (data not shown). In contrast, 100 μg DEHP did not show the enhancing effects (Figure 2A,E).

### DEHP modulates the protein expression of chemokines in the skin related to Dp

**Figure 3:**
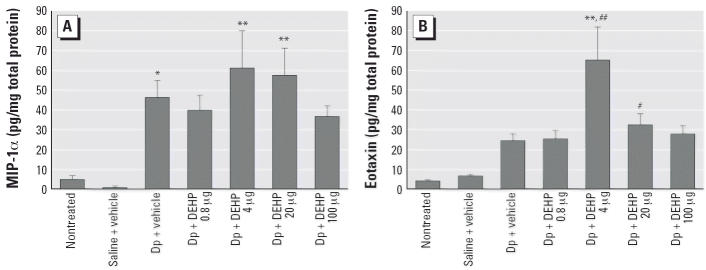
Effects of DEHP on the protein expression of chemokines MIP-1α (*A*) and eotaxin (*B*) in ear homogenates 48 hr after the last injection of Dp as determined by ELISA. Differences among groups were determined using Dunnett’s multiple comparison test; data shown are the mean ± SE of eight animals per group. *p < 0.05 vs. nontreated group and saline + vehicle group. **p < 0.01 vs. nontreated group and saline + vehicle group. ^#^*p* < 0.05 vs. nontreated group. ^##^*p* < 0.01 vs. Dp + vehicle group.

Treatment with Dp increased the expression of MIP-1α (Figure 3A) and eotaxin (Figure 3B) compared with nontreated or saline treated groups (*p* < 0.05 for MIP-1α; not significant for eotaxin). In the presence of allergen, exposure to 4 or 20 μg DEHP increased the expression of these chemokines compared with no treatment (*p* < 0.01 for MIP-1α after treatment with 4 or 20 μg DEHP; *p* < 0.01 for eotaxin after treatment with 4 μg DEHP; *p* < 0.05 for eotaxin after treatment with 20 μg DEHP). Furthermore, combined exposure to allergen and DEHP enhanced the protein expression of eotaxin compared with exposure to allergen and vehicle (*p* < 0.01 for 4 μg DEHP; not significant for 20 μg DEHP).

## Discussion

In the present study we have shown that exposure to DEHP at a dose of 0.8–20 μg caused deterioration of atopic dermatitis-like skin lesions related to mite allergen (Dp) in NC/Nga mice, which is evidenced by macroscopic and microscopic examinations. However, in animals treated with 100 μg DEHP, we did not observe significant effects. Furthermore, these enhancing effects are paralleled by the expression of proinflammatory molecules such as eotaxin and MIP-1α in ear tissue in overall trend.

DEHP has been widely used in polyvinyl chloride and other plastics, including building products, clothing, food packing, children’s products, and media devices. Thus, the general population can be exposed to DEHP in food, water, and air via ingestion or inhalation. Jaakkola et al. (1999) suggested that DEHP is associated with the development of asthma in children. In addition, DEHP has been shown to possess adjuvant activity for allergen-related IgG1 response in mice (Larsen et al. 2001). Several reports have shown that DEHP in headphones (Walker et al. 2000) and in a polyvinyl chloride grip on cotton gloves (Sugiura et al. 2000, 2002) can induce cases of contact urticaria syndrome. However, the association between DEHP and atopic dermatitis has never been elucidated.

In the present study, DEHP caused deterioration of skin lesions and chemokine expression related to Dp in atopic subjects (NC/Nga mice) at doses about 1,000-fold lower than the no observed adverse effect level that was determined on the basis of histologic changes in the liver of rodents (19 mg/kg/day) (Carpenter et al. 1953). Furthermore, we also evaluated the effects of DEHP on an atopic dermatitis model using a different strain, BALB/c mice; the results on BALB/c mice paralleled those on NC/Nga mice in overall trend (data not shown). The active doses used in our study are comparable to the recently calculated daily intake, the reference dose of the U.S. Environmental Protection Agency [20 μg/kg/day (U.S. EPA 2006)], and the tolerable daily intake value settled by the European Union Scientific Committee for Toxicity [50 μg/kg/day (European Union Scientific Committee for Toxicity 2002)] in humans. These reports and our results suggest that ambient exposure to DEHP is a possible contributor to the recent increase in the incidence of atopic dermatitis.

The present study showed that exposure to 0.8–20 μg DEHP caused deterioration of atopic dermatitis-like skin lesions related to Dp; however, exposure to 100 μg DEHP did not show the enhancing effects. The similar results (inverted U-shaped dose response) have been typically shown with the effects of endocrine-disrupting chemicals. Octylphenol and 4-*n*-nonylphenol are among the most important alkylphenolic compounds widely used in industry and appear to possess intrinsic estrogenic activity (White et al. 1994). Exposure to these environmental chemicals has reportedly exhibited an inverted U-shaped dose response on the number of unshelled embryos of snails (Duft et al. 2003). In addition, bisphenol A, an environmental endocrine disruptor, has dose-dependently increased the mRNA levels for aryl hydrocarbon receptor in mouse gonads at a dose of 0.02–200 μg/kg/day but decreased them at higher-doses (Nishizawa et al. 2005). DEHP has also been reported as an endocrine-disrupting chemical in the United States and Europe (Dalgaard et al. 2000; Tandon et al. 1991). Our results might indicate an association between the exposure to endocrine-disrupting chemicals and the aggravation of allergic responses.

Infiltration of inflammatory cells into tissues is regulated by chemokines. Eotaxin is an important contributor to eosinophil recruitment in atopic dermatitis. The levels of eotaxin are significantly higher in patients with atopic dermatitis than in healthy people (Jahnz-Rozyk et al. 2005). Gene expression for eotaxin is up-regulated in ovalbumin-sensitized skin sites (Spergel et al. 1999). In contrast, MIP-1α has been chemotactic for neutrophils, macrophages, T cells, and B cells, affecting their activation. Spontaneous production of MIP-1α is augmented in atopic dermatitis patients (Kaburagi et al. 2001). An increased transcription level for MIP-1α has been associated with atopic dermatitis (Xin et al. 2001). In the present study, exposure to 4 or 20 μg DEHP in the presence of allergen increased the expression of these chemokines compared with vehicle combined with allergen. The results suggest that the enhancement of the expression of these chemokines by DEHP might, at least partially, play an important role in that of atopic dermatitis related to Dp. However, results on the protein expression of these chemokines 48 hr after the last allergen instillation were slightly different from those on the clinical scores 24 hr after the instillation. The expression of these molecules might be enhanced at the earlier time points. We plan to investigate the time course of the protein expression of these molecules in the next stage of our research.

In conclusion, DEHP, which is widely used in plasticized products and ubiquitous in the developed world, can be responsible for the recent increase in atopic dermatitis. The enhancing effects may be mediated through the enhanced expression of chemokines.
