# Supplementary material for: Di-(2-ethylhexyl) Phthalate Enhances Atopic Dermatitis-Like Skin Lesions in Mice
Source: Environ Health Perspect. 2006 May 15;114(8):1266–9. doi: 10.1289/ehp.8985 (PMC1552025; doi:10.1289/ehp.8985)
Supplement: Supplemental Figures and Tables [file ehp0114-001266s1.pdf]

Supplemental Material

Hirohisa Takano[1,2], Rie Yanagisawa[1], Ken-ichiro Inoue[1], Takamichi Ichinose[3], Kaori Sadakane[3], Toshikazu Yoshikawa[2]

1: Environmental Health Sciences Division, National Institute for Environmental Studies, Tsukuba, Japan.

2: Inflammation and Immunology, Kyoto Prefectural University of Medicine, Kyoto, Japan.

3: Department of Health Sciences, Oita University of Nursing and Health Sciences, Oita, Japan.

Address for correspondence:

Hirohisa Takano, M.D., Ph.D.

Environmental Health Sciences Division

National Institute for Environmental Studies

16-2 Onogawa, Tsukuba, 305-8506, Japan.

tel: +81-298-50-2336, fax: +81-298-50-2334

e-mail: htakano@nies.go.jp

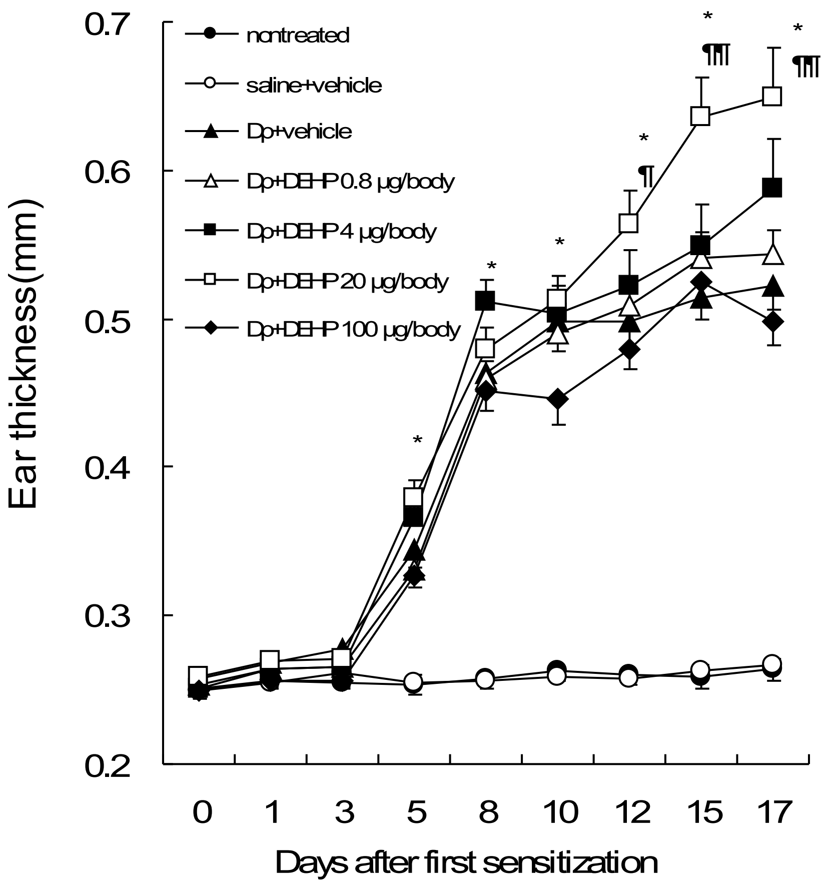

Figure E1

Exposure to DEHP exacerbates atopic dermatitis–like skin lesions induced by mite allergen (Dp). We evaluated ear thickening 24 hours after each injection. Differences among groups were determined using Dunnet's multiple comparison test. Data are the means ± SEM of 16 animals per group.

*; p<0.01: Dp treated groups vs. nontreated group and saline+vehicle group

¶; p<0.05: Dp+DEHP 20 µg/body group vs. Dp+vehicle group

¶¶; p<0.01: Dp+DEHP 20 µg/body group vs. Dp+vehicle group
